# Temporal and spatial dynamics of gastrointestinal parasite infection in Père David’s deer

**DOI:** 10.7717/peerj.11335

**Published:** 2021-05-05

**Authors:** Shanghua Xu, Shumiao Zhang, Xiaolong Hu, Baofeng Zhang, Shuang Yang, Xin Hu, Shuqiang Liu, Defu Hu, Jiade Bai

**Affiliations:** 1 School of Ecology and Nature Conservation, Beijing Forestry University, Beijing, China; 2 Department of Research, Beijing Milu Ecological Research Center, Beijing, China; 3 College of Animal Science and Technology, Jiangxi Agricultural University, Nanchang, Jiangxi, China; 4 Beijing Key Laboratory of Captive Wildlife Technologies, Beijing Zoo, Beijing, China

**Keywords:** Père David’s deer, Gastrointestinal parasites, Temporal and spatial variation, Reproductive period

## Abstract

**Background:**

The Père David’s deer (*Elaphurus davidianus*) population was established from only a small number of individuals. Their genetic diversity is therefore relatively low and transmissible (parasitic) diseases affecting them merit further attention. Parasitic infections can affect the health, survival, and population development of the host. However, few reports have been published on the gastrointestinal parasites of Père David’s deer. The aims of this study were: (1) to identify the intestinal parasites groups in Père David’s deer; (2) to determine their prevalence and burden and clarify the effects of different seasons and regions on various indicators of Père David’s deer intestinal parasites; (3) to evaluate the effects of the Père David’s deer reproductive period on these parasites; (4) to reveal the regularity of the parasites in space and time.

**Methods:**

In total, 1,345 Père David’s deer faecal samples from four regions during four seasons were tested using the flotation (saturated sodium nitrate solution) to identify parasites of different genus or group, and the McMaster technique to count the number of eggs or oocysts.

**Results:**

Four groups of gastrointestinal parasites were found, of which strongyles were dominant; their prevalence and burden were significantly higher than other groups. Significant temporal and spatial effects on gastrointestinal parasitic infection were found. Parasite diversity, prevalence, parasite burden, and aggregation were the highest in summer. Among the four regions, parasite diversity, prevalence, and burden were the highest in the Dongting Lake area. In addition, parasite diversity and burden during the reproductive period of Père David’s deer was significantly higher than during the post-reproductive period.

**Conclusions:**

The summer season and the reproductive period of Père David’s deer had great potential for parasite transmission, and there is a high risk of parasite outbreaks in the Dongting Lake area.

## Introduction

Parasites have a negative impact on the health of the host and may affect all parts of the body (Rauque et al., 2011). Parasites can cause physiological stress, malnutrition, tissue damage, reduced reproduction, weakened adaptability, and even directly lead to death (Thomas et al., 1996; Williams, Poulin & Sinclair, 2004; Fredensborg & Poulin, 2006; Hideko Tatakihara et al., 2008; Poulin, 2010). Thus, parasites could play a role in regulating animal population numbers (Anderson & May, 1978). Previous research has shown that parasites can harm Cervidae: wild roe deer lose weight or may even die because of high parasite loads (Maublanc et al., 2009); and abomasal parasite syndrome caused by parasites leads to approximately 84% mortality in North American elk (Woodbury & Parry, 2009); progressive hair loss, emaciation, weakness, debilitation, and death occurred in numerous black-tailed deer caused by parasites (Foreyt, Hall & Bender, 2004).

The Père David’s deer *(Elaphurus davidianus)* is one of the rarest and most endangered deer species worldwide (Han et al., 2016). The Père David’s deer was once widely distributed along the Yangtze and Yellow Rivers (Hu & Jiang, 2002). Owing to anthropogenic and natural pressures, such as hunting, rapid habitat reduction, and climate change (Bunert et al., 2018; Zhang et al., 2018), it became extinct in the wild in China, during the early twentieth century (Ohtaishi & Gao, 1990). Fortunately, the Père David’s deer had been introduced into Europe (Jiang, Kaji & Ping, 2016). By the beginning of the nineteenth century, there were only 18 individual Père David’s deer worldwide. The current Père David’s deer populations are all descended from these individuals (Jones, 1951). Since 1985, Père David’s deer have been reintroduced into China (Jiang, 2000; Jiang et al., 2000). By the end of 2018, after more than 30 years of breeding and expansion, the number of Père David’s deer had grown to nearly 7,000, forming three large populations in Dafeng (Jiangsu), Shishou (Hubei), and the Dongting Lake area (Hunan) and spreading to over 100 locations throughout the country (Wang et al., 2020).

Although the Père David’s deer population has increased, it was established from only a few individuals; therefore, their genetic diversity is relatively low. There was a genetic bottleneck in the inbreeding population (Zeng et al., 2013). It is possible that unforeseen factors, especially infectious diseases could fatally impact the Père David’s deer population. They are affected by season (Tahir et al., 2016) and climatic conditions, such as temperature (Podder, Gupta & Saha, 2009) and rainfall (Klaus et al., 2018), which play important roles in their transmission and infection. In addition, the physical and reproductive condition of the host changes seasonally (Gaspar-López et al., 2010) because the quality and availability of their resources (such as energy and protein) for maintenance, growth, reproduction, and lactation are seasonal (Lochmiller & Deerenberg, 2000). Parasite dynamics can be affected by abiotic factors (such as season and climatic conditions); for example, precipitation caused significant changes in the gastrointestinal parasites of eastern chimpanzees (Gillespie et al., 2010) and temperature had a significant impact on the transmission of parasites in the gastrointestinal tracts of Arctic ungulates (Kafle et al., 2018). Alpine chamois and red deer parasite burden was affected by various biotic factors, including reproduction and immune status (Corlatti et al., 2012; De la Peña et al., 2020a). The host often faces a life history trade-off between reproduction and immunity (Corlatti et al., 2012; De la Peña et al., 2020a).

There are a few recent reports on Père David’s deer parasites; however, these ones are mainly on intracellular parasites (Han et al., 2016; Huang et al., 2020; Xie et al., 2019; Zhang et al., 2015), whereas the gastrointestinal parasites of Père David’s deer have gained little attention. Under the premise that the Père David’s deer population was established from only a few individuals, the stability of the population may be relatively poor. Therefore, it is particularly important to prevent the spread of diseases, including parasites, in Père David’s deer. To date, the temporal and spatial trends of parasites in the gastrointestinal tract of Père David’s deer are unclear. Thus, the aims of this study were to identify the intestinal parasites groups in Père David’s deer, to determine their prevalence and burden and clarify the effects of different seasons and regions on various indicators of Père David’s deer intestinal parasites, to evaluate the effects of the Père David’s deer reproductive period on these parasites, and reveal the regularity of the parasites in space and time. The results will help us understand the times and sites at which the Père David’s deer are most at risk, and to manage them appropriately to prevent outbreaks of parasitic diseases when necessary.

## Material and Methods

### Study sites and sample collection

**Figure 1:**
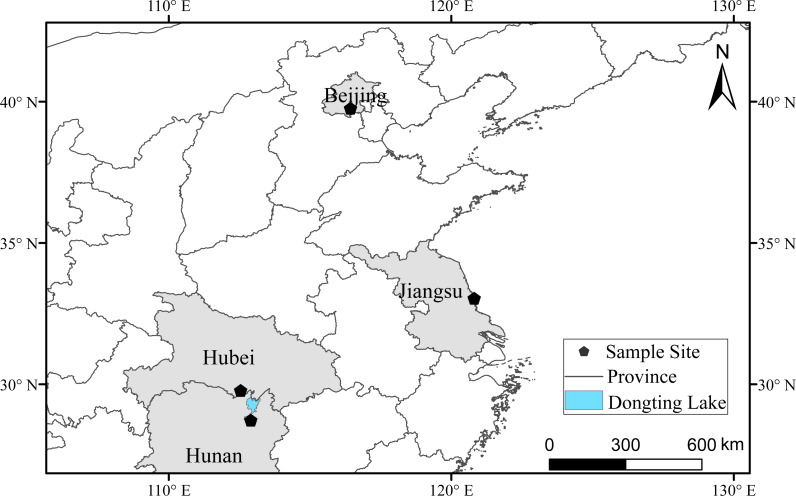
Sample collection sites. Beijing indicates Beijing Milu Ecological Research Centre; Shishou indicates Hubei Shishou Milu National Nature Reserve; Dongting indicates The East Dongting Lake National Nature Reserve; Dafeng indicates Jiangsu Dafeng Milu National Nature Reserve.

The four sampling locations were as follows: (1) Beijing Milu Ecological Research Centre located in the northern part of the North China Plain and the southern edge of the Yanshan Mountains. It belongs to Daxing District in the southern suburb of Beijing and is hereinafter referred to as ‘Beijing’. (2) Hubei Shishou Milu National Nature Reserve located at the geographical centre of the middle reaches of the Yangtze River (Jianghan Plain, Dongting Lake Plain). It belongs to Shishou City, Hubei Province, and is hereinafter referred to as ‘Shishou’. (3) The East Dongting Lake National Nature Reserve located in the northeast of Hunan Province and northeast of the Yangtze River. It belongs to Yueyang City, Hunan Province, and is hereinafter referred to as ‘Dongting’. (4) Jiangsu Dafeng Milu National Nature Reserve located on the coast of the Yellow Sea, east of Jiangsu Province, south of Yancheng City, and east of the Yellow Sea. It belongs to Yancheng City, Jiangsu Province, and is hereinafter referred to as ‘Dafeng’. The locations are shown in Fig. 1.

The Père David’s deer roam freely in these preserves. There are rivers, lakes, and other wetlands in their habitats. Water and food are freely available. Their diet mainly consists of plants, including *Spartina alterniflora, Suaeda spp., Spartina anglica, Imperata cylindrical, Phragmites australis,* and *Scirpus triqueter (Scirpus triqueter* L.) (Wu et al., 2011).

In spring, we collected 200 Père David’s deer faecal samples in Beijing and 100 in Shishou; in summer, we collected 200, 96, 95, and 96 samples in Beijing, Shishou, Dongting, and Dafeng, respectively; in autumn, we collected 192 and 90 samples in Beijing and Shishou, respectively; and in winter, we collected 176 and 100 samples in Beijing and Shishou, respectively (the details are shown in Table 1). At 19:00 the day before collection, all fresh faeces in five randomly divided 20 m × 20 m squares were cleaned away so that they would not interfere with the faeces collected the next day. Each fresh faecal sample was collected using a sterile disposal latex glove (Beijing Huateng Rubber Plastic & Latex Products Co., Ltd, Beijing, China) and placed in a 50-mL sterile conical centrifuge tube (Thermo Fisher Scientific, Beijing, China) containing 10% formalin solution (Formalin could greatly slow down or prevent the growth of parasites and the hatching of eggs (Bowman, 2008), thereby reducing the error of egg counting during transportation and experiments. But fixation would affect subsequent identification of parasites. This is a flaw in the research. Future research would collect a part of duplicate samples, one part would be fixed for egg counting, and the other part would be used for hatching eggs and identifying species). The solid–liquid ratio of faecal matter to formalin solution was 1:4. The samples were shipped to the laboratory (Laboratory of Non-invasive Research Technology for Endangered Species, College of Nature Conservation, Beijing Forestry University) and stored at room temperature until further processing.

**Table 1:** Number of faecal samples collected from four regions in four seasons.

Sampling sites	Sampling time				Total
	Spring	Summer	Autumn	Winter	
Beijing	200	200	192	176	768
Shishou	100	96	90	100	386
Dongting	–	95	–	–	95
Dafeng	–	96	–	–	96
Total	300	487	282	276	1345

**Notes.**

The “–” indicates there was no sample collected.

### Parameters

The four measurements of parasites defined in this study were: (1) Prevalence, the number of hosts infected with one or more individuals of a particular parasitic species (or taxonomic group), divided by the number of hosts examined for that parasitic species (Bush et al., 1997). (2) Parasite burden was estimated as the number of a parasitic form (oocysts, eggs or larvae) in a single faecal sample (Bush et al., 1997; De la Peña et al., 2020a). In this study the parasite burden was assessed by calculating the faecal oocyst or egg counts. (3) Diversity, the richness of parasite groups (S), reflecting the number of parasite groups per deer (Hu et al., 2018). (4) Parasite aggregation, the degree of non-uniformity in the distribution of the parasites in infected hosts (Poulin, 1993). Here, we used the corrected moment estimate of K (where K provides an inverse measure of the degree of parasite aggregation (Gregory & Woolhouse, 1993) to indicate the degree of aggregation), as per the equation used by Sherrard-Smith et al. (2015):

\begin{eqnarray*}K= \frac{({x}^{2}- \frac{{\sigma }^{2}}{N} )}{({\sigma }^{2}-x)} \end{eqnarray*}

where x, *σ*^2^, and N represent mean parasite burden, variance, and sample size, respectively. K is an inverse measure of aggregation. K is the most commonly used measure because the corrected moment estimate of K varies less than other indicators when faced with different mean parasite loads and sample sizes (Sherrard-Smith et al., 2015).

### Parasitological analyses

Saturated sodium nitrate solution floatation method was used to identify parasite groups. Parasite groups (strongyles, *Eimeria* spp., *Moniezia* spp., and *Trichuris* spp.) were identified based on the criteria given by Bowman (2008). It was impossible to determine the exact genus of the strongyle-type parasites based on the eggs (Bowman, 2008). The group of strongyles include superfamilies Strongyloidea, Trichostrongyloidea, and Ancylostomatoidea (Bowman, 2008). Therefore, the “strongyles” was used to refer them in this article. When a more specific diagnosis is required, it is necessary to culture the stages present in the faeces to the infective stage(third-stage) (Bowman, 2008). But in order to reduce the difference caused by the hatching of the eggs during the experiment (the inspection time is sequential), the faeces were soaked by formalin, so the hatching identification cannot be done. Follow-up research was needed.

The Master technique was used for quantitative analysis (Hu et al., 2016). Faecal samples (2 g) were suspended in 58-mL sodium nitrate saturated solution (specific gravity 1.2) and stirred continuously for 20 min to homogenise the mixture. Large plant debris were removed via 0.18-mm mesh. The remaining homogenised aliquot of the filtrate was transferred into both McMaster counting chambers (Shanghai Veterinary Research Institute, Chinese Academy of Agricultural Sciences). Counting was performed under a light-optical microscope at 100 × magnification, started 5 min after loading the slide. The eggs per gram were calculated as: Eggs per gram = [n/(0.15 × 2)] × V/m, where n is the mean number of eggs or oocysts in the two counting chambers, 0.15 is the volume of each counting chamber, and there are two chambers, and V and m are the volume of the homogenised faecal sample and weight of the faeces, respectively; in this case *V* = 60 mL and *m* = 2 g (Hu et al., 2016) . The faecal egg density was estimated using a modified McMaster technique with a sensitivity of 100 eggs per gram.

### Statistical analyses

The seasons were defined as follows: spring (March–May); summer (June–August); autumn (September–November); and winter (December–February). Previous studies have identified that the reproductive behavior, sex hormones of Père David’s deer and concluded that reproduction, breastfeeding, and infant care behaviours are predominant during late May to August in late spring and summer (Li et al., 2001). For this reason, in this study we defined the reproductive period as June-August, and the post-reproductive period as March–May, September–November and December–February.

The difference of parasite prevalence between the groups was detected by a generalized linear model (binomial distribution, logit link), and the dependent variable was the prevalence of each group (strongyles, *Eimeria* spp., *Moniezia* spp., and *Trichuris* spp.), and the factor and main effect are the “group” (1,2,3,4, data format was shown in the supplementary file). The difference of parasite burden between the groups was detected by a generalized linear model (negative binomial distribution, log link), and the dependent variable was the burden of each group, the factor and main effect are the “group”.

The difference of parasite diversity (the number of a sample which has one or two or three or four species of parasites) between seasons and regions was detected using Poisson distribution (identity link) by a generalized linear mixed model (GLMM). Selected the variable “parasite diversity” as the dependent variable; seasons or regions as fixed effects; sample ID and microhabitat (where samples were found, included grass, bare land, waterside and mud puddle) as random effects. The effects of seasons and regions on the prevalence were investigated using Binomial distribution (logit link) generalized linear mixed models. Selected the variable “parasite prevalence” as the dependent variable; seasons or regions as fixed effects; sample ID and microhabitat as random effects. Comparison of two seasons or regions uses pairwise comparison in generalized linear mixed model. The burden was assessed using linear mixed models (LMM, with log (*x* + 1) transformed burden data, because some samples have zero burden data). Selected “burden” as dependent variable; seasons or regions as fixed effects; microhabitats as random effects. The two-sample Kolmogorov–Smirnov test was applied to test the variation of distributions (aggregation) among the sample four parasite groups. All statistical analyses were performed using SPSS ver. 26.0 software (IBM Corp., Armonk, NY, USA).

**Figure 2:**
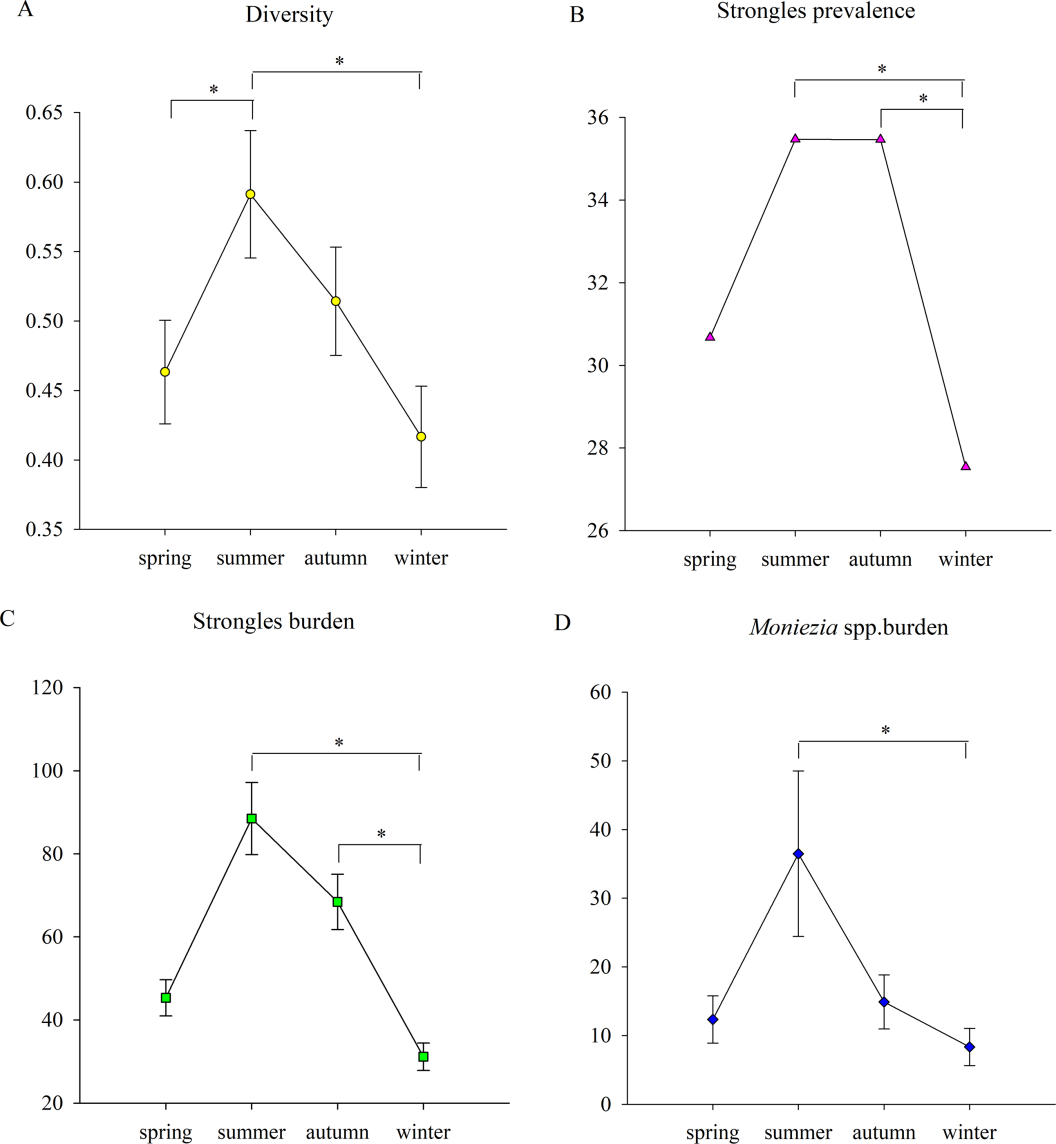
The seasonal trends of parasite parameters. (A) The seasonal trends of parasite diversity; (B) Strongyles prevalence; (C) the Strongyles burden; (D) the *Eimeria* spp. burden; (E) the *Moniezia* spp. burden; (F) the *Trichuris spp.* burden. An asterisk (*) means significant differences.

## Results

Four groups of parasites were identified from the 1,345 faecal samples: strongyles, *Eimeria* spp., *Moniezia* spp., and *Trichuris* spp. The percentage of non-parasitised samples was 57.03%. The parasitised samples included samples in which one (*S* = 1), two (*S* = 2), three (*S* = 3), or all four (*S* = 4) parasite groups were identified. Their percentages were 31.08%, 10.63%, 0.89%, and 0.37%, respectively. The strongyles group was the dominant parasite group, with higher prevalence (GLM, *χ*^2^ = 539.135, *df* = 3, *p* < 0.001) and burden (GLM, *χ*^2^ = 6091.120, *df* = 3, *p* < 0.001) than those of the other groups.

**Figure 3:**
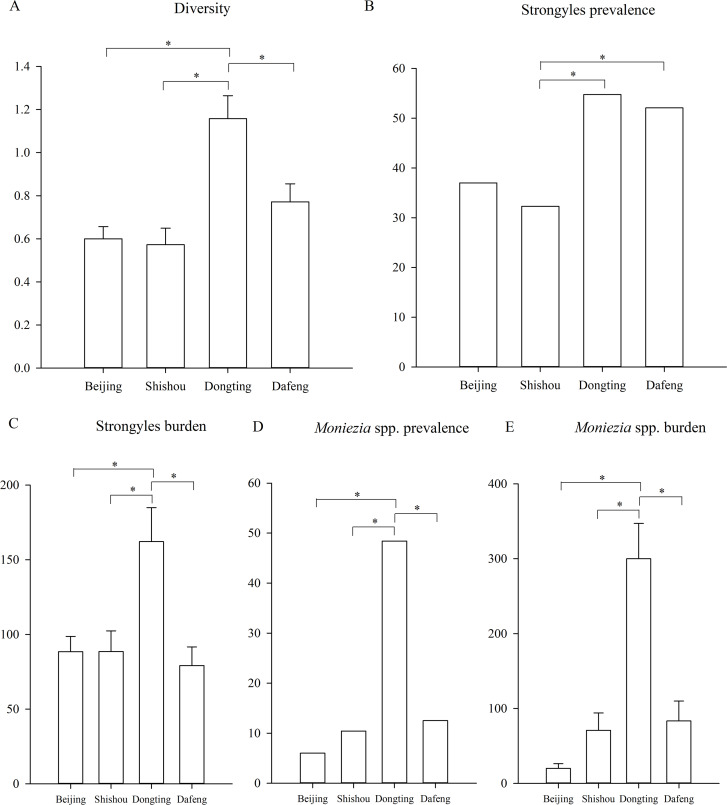
Differences between the regions. (A) The difference of the parasite diversity between the regions; (B) the Strongyles prevalence; (C) the Strongyles burden; (D) the *Moniezia* spp. prevalence; (E) The *Moniezia* spp. burden between the regions. An asterisk (*) means significant differences.

Parasite diversity was highest in summer and lowest in winter (GLMM, *F* = 3.399, df1 = 3, df2 = 1150, *p* = 0.017) (Fig. 2A). The statistical values and model effect results are shown in Table 2. Summer had significantly higher diversity than spring or winter (GLMM, *Z* = 2.149, *df* = 1150, *p* = 0.032, summer-spring; *Z* = 3.014, *df* = 1150, *p* = 0.003, summer-winter). The highest parasite diversity was observed in Dongting while the lowest occurred in Shishou (GLMM, *F* = 7.924, df1 = 3, df2 = 483, *p* < 0.001) (Fig. 3A). The parasite diversity of Dongting was significantly higher than Beijing, Shishou and Dafeng (GLMM, *Z* = 4.527, *df* = 483, *p* < 0.001, site Dongting- Beijing; *Z* = 4.341, *df* = 483, *p* < 0.001, site Dongting- Shishou; GLMM, *Z* = 2.722, *df* = 483, *p* = 0.007, site Dongting- Dafeng).

**Table 2:** The diversity between different seasons and regions.

Factors	Sample number	Diversity	GLMM				
			Effects				p
Spring	300	0.4633 ± 0.03727	Fixed	F	df (n)	df (d)	
Summer	296	0.5912 ± 0.04585		3.399	3	1150	0.017
Autumn	282	0.5142 ± 0.03899	Random	Estimate	Residual	Z	
Winter	276	0.4167 ± 0.03649	1	0.014	0.040	0.343	0.732
Total	1154		2	0.001	0.002	0.458	0.647
Beijing	200	0.6000 ± 0.05627	Fixed	F	df (n)	df (d)	
Shishou	96	0.5729 ± 0.07658		7.924	3	483	<0.001
Dongting	95	1.1579 ± 0.10616	Random	Estimate	Residual	Z	
Dafeng	96	0.7708 ± 0.08440	1	0.382	0.355	1.076	0.282
Total	487		2	0.058	0.182	0.319	0.749

There were no significant seasonal differences in strongyle prevalence (GLMM, *F* = 1.946, df1 = 3, df2 = 1150, *p* = 0.120) (Fig. 2B). The highest and lowest strongyle burden appeared in summer and winter (LMM, *F* = 3.747, df1 = 3, df2 = 1150, *p* = 0.011) (Fig. 2C). A significantly higher burden was observed in summer and autumn than in winter (LMM, *Z* = 2.948, *df* = 1150, *p* = 0.003 summer- autumn; LMM, *Z* = 2.565, *df* = 1150, *p* = 0.010, autumn-winter). Summer and winter had the highest and lowest levels of strongyle aggregation (K-W, *H* = 14.229, *df* = 3, *p* = 0.003). The aggregation in summer was significantly higher than winter (K-S, *Z* = 2.194, *n* = 572, *p* < 0.001).

Regionally, the highest strongyle prevalence was observed in Dongting and the lowest in Shishou (GLMM, *F* = 5.208, df1 = 3, df2 = 483, *p* = 0.002) (Fig. 3B). The statistical values and model effect results are shown in Table 3. Dongting had a higher strongyle prevalence than Beijing or Shishou (GLMM, *Z* = 2.875, *df* = 483, *p* = 0.004, Dongting-Beijing; *Z* = 3.198, *df* = 483, *p* = 0.001, Dongting- Shishou), and Dafeng had a higher prevalence than Beijing or Shishou (GLMM, *Z* = 2.448, *df* = 483, *p* = 0.015, Dongting-Beijing; *Z* = 2.822, *df* = 483, *p* = 0.005, Dafeng- Shishou). Dongting and Dafeng yielded the highest and lowest strongyle parasite burden (LMM, *F* = 5.142, df1=3, df 2= 483, *p* = 0.002) (Fig. 3C). A significantly higher burden was found in Dongting than in Beijing or Shishou (LMM, *Z* = 3.350, *df* = 483, *p* = 0.001, Dongting-Beijing; *Z* = 3.276, *df* = 483, *p* = 0.001, Dongting- Shishou). Dafeng had a higher burden than Shishou (LMM, *Z* = 1.985, *df* = 483, *p* = 0.048). The highest and lowest levels of aggregation were displayed in Beijing and Dongting (K-W, *H* = 15.495, *df* = 3, *p* = 0.001), with a higher level in Beijing than in Dongting (K-S, *Z* = 1.930, *n* = 295, *p* = 0.001), and a higher level in Shishou than in Dongting (K-S, *Z* = 1.551, *n* = 191, *p* = 0.016).

There were no significant differences in prevalence (GLMM, *F* = 0.714, df1=3, df2=1150, *p* = 0.544, seasons; GLMM, *F* = 0.665, df1=3, df2=483, *p* = 0.574, regions), burden (LMM, *F* = 1.011, df1=3, df2=1150, *p* = 0.387, seasons; LMM, *F* = 0.825, df1=3, df2=483, *p* = 0.481, regions) or aggregation (K-W, *H* = 2.954, *df* = 3, *p* = 0.399, seasons; K-W, *H* = 2.420, *df* = 3, *p* = 0.490, regions) of *Eimeria* spp. between seasons or regions. There were no significant differences in prevalence (GLMM, *F* = 0.459, df1=3, df2=1150, *p* = 0.711, seasons; GLMM, *F* = 0.030, df1=3, df2=483, *p* = 0.993, regions), burden (LMM, *F* = 1.857, df1=3, df2=1150, *p* = 0.135, seasons; LMM, *F* = 0.034, df1=3, df2=483, *p* = 0.992, regions) or aggregation (K-W, *H* = 5.313, *df* = 3, *p* = 0.150, seasons; K-W, *H* = 0.247, *df* = 3, *p* = 0.970, regions) of *Trichuris* spp. between seasons or regions.

**Table 3:** The prevalence and burden of Strongyles in different seasons and different regions.

Factors	Sample number	Strongyles Prevalence (%)	GLMM					Strongyles burden	LMM				
			Effects				*p*		Effects				*p*
Spring	300	30.67	Fixed	F	df (n)	df (d)		45.33 ± 4.36	Fixed	F	df (n)	df (d)	
Summer	296	35.47		1.946	3	1150	0.120	88.51 ± 8.65		3.747	3	1150	0.011
Autumn	282	35.46	Random	Estimate	Residual	Z		68.44 ± 6.67	Random	Estimate	Residual	Z	
Winter	276	27.54	1	0.050	0.365	0.136	0.892	31.16 ± 3.31	1	0.019	0.074	0.258	0.797
			2	0.020	0.212	0.094	0.925		2	0.053	0.012	0.398	0.691
Beijing	200	37	Fixed	F	df (n)	df (d)		88.50 ± 10.23	Fixed	F	df (n)	df (d)	
Shishou	96	32.29		5.208	3	483	0.002	88.54 ± 13.88		5.142	3	483	0.002
Dongting	95	54.74	Random	Estimate	Residual	Z		162.11 ± 22.85	Random	Estimate	Residual	Z	
Dafeng	96	52.08	1	0.340	0.282	0.120	0.904	79.17 ± 12.47	1	0.037	0.007	0.514	0.607
			2	0.015	0.193	0.078	0.938		2	0.079	0.042	0.189	0.850

There were no significant seasonal differences in *Moniezia* spp. prevalence (GLMM, *F* = 0.715, df1=3, df2=1150, *p* = 0.543), burden (LMM, *F* = 1.973, df1=3, df2=1150, *p* = 0.116) and aggregation (K-W, *H* = 4.724, *df* = 3, *p* = 0.193).

The highest and lowest prevalence of *Moniezia* spp. were observed in Dongting and Beijing respectively (GLMM, *F* = 22.979, df1=3, df2=483, *p* < 0.001, regions) (Fig. 3D). The statistical values and model effect results are shown in Table 4. Dongting had a higher *Moniezia* spp. prevalence than Beijing, Shishou or Dafeng (GLMM, *Z* = 7.673, *df* = 483, *p* < 0.001, Dongting- Beijing; *Z* = 6.292, *df* = 483, *p* < 0.001, Dongting- Shishou; *Z* = 5.839, *df* = 483, *p* < 0.001, Dongting- Dafeng). The highest *Moniezia* spp. burden was observed in Dongting and the lowest in Beijing (LMM, *F* = 36.289, df1=3, df2=483, *p* < 0.001) (Fig. 3E). A significantly higher burden was found in Dongting than in Beijing, Shishou, or Dafeng (LMM, *Z* = 10.152, *df* = 483, *p* < 0.001, Dongting- Beijing; *Z* = 7.633, *df* = 483, *p* < 0.001, Dongting- Shishou; *Z* = 7.281, *df* = 483, *p* < 0.001, Dongting- Dafeng). The highest and lowest levels of aggregation were displayed in Shishou and Dongting (K-W, *H* = 89.411, *df* = 3, *p* < 0.001), with a higher level in Beijing than in Dongting (K-S, *Z* = 3.404, *n* = 295, *p* < 0.001), and a higher level in Shishou than in Dongting (K-S, *Z* = 2.626, *n* = 191, *p* < 0.001), a higher level in Dafeng than in Dongting (K-S, *Z* = 2.482, *n* = 191, *p* < 0.001).

**Table 4:** The prevalence and burden of *Moniezia* spp. in different seasons and different regions.

Factors	Sample number	*Moniezia* spp.	GLMM					*Moniezia* spp.	LMM				
		Prevalence (%)	Effects				*p*	Burden	Effects				*p*
Spring	300	4.33	Fixed	F	df (n)	df (d)		12.33 ± 3.44d	Fixed	F	df (n)	df (d)	
Summer	296	7.43		0.715	3	1150	0.543	36.49 ± 12.05		1.973	3	1150	0.116
Autumn	282	5.67	Random	Estimate	Residual	Z		14.89 ± 3.92	Random	Estimate	Residual	Z	
Winter	276	3.99	1	0.045	0.005	0.895	0.371	8.33 ± 2.69	1	0.042	0.137	0.310	0.757
			2	0.022	0.071	0.307	0.759		2	0.053	0.012	0.452	0.651
Beijing	200	6.00	fixed	F	df (n)	df (d)		20.00 ± 6.22	fixed	F	df (n)	df (d)	
Shishou	96	10.42		22.979	3	483	<0.001	70.83 ± 23.18		36.289	3	483	<0.001
Dongting	95	48.42	Random	Estimate	Residual	Z		300.00 ± 47.25	Random	Estimate	Residual	Z	
Dafeng	96	12.50	1	0.001	0.059	0.016	0.987	83.33 ± 26.56e	1	0.071	0.063	1.127	0.260
			2	0.080	0.111	0.718	0.473		2	0.015	0.193	0.078	0.938

**Figure 4:**
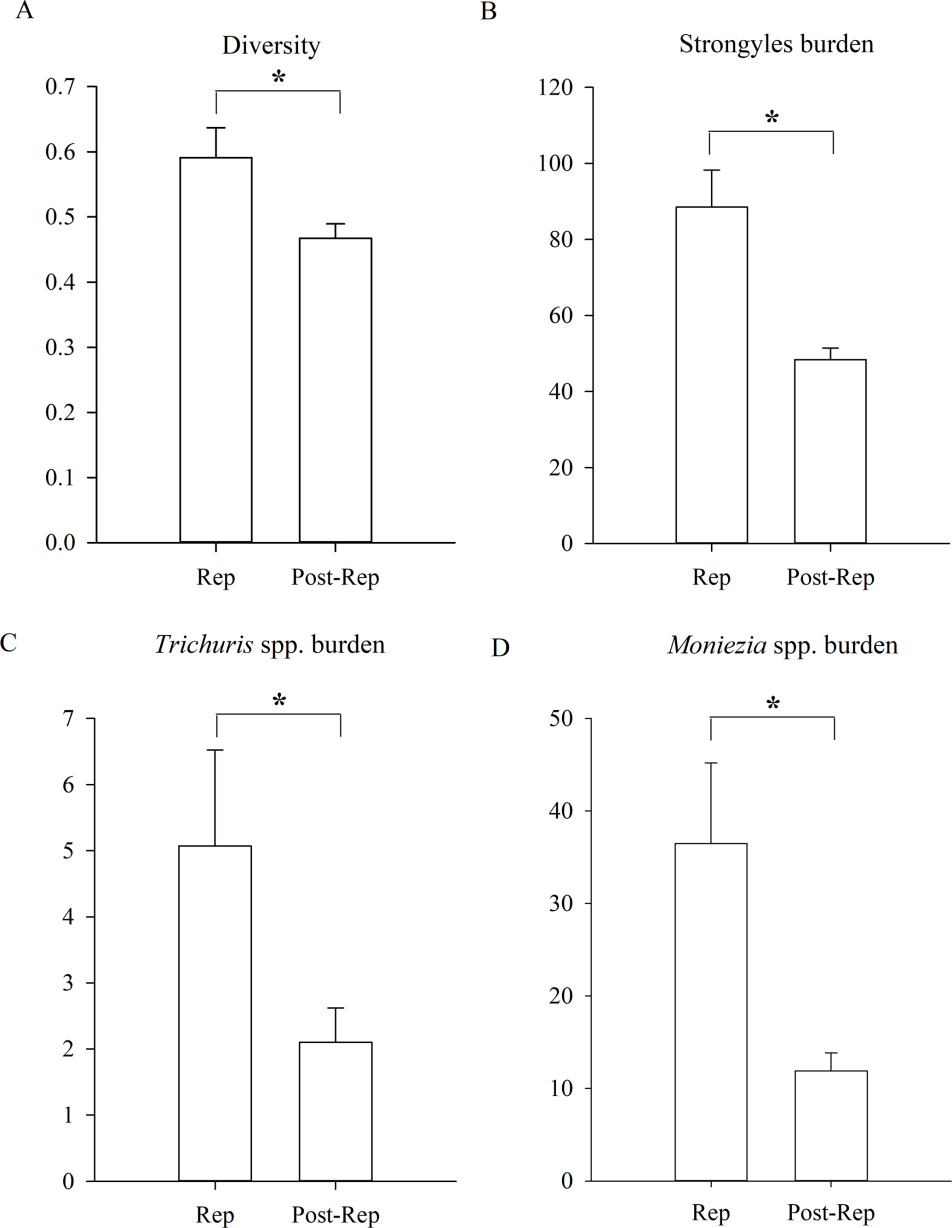
Differences between reproductive period of and post-reproductive period. (A) The parasite diversity differences; (B) the Strongyles burden differences; (C) the Eimeria spp. burden differences; (D) the *Moniezia* spp. burden differences; (E) the *Trichuris spp.* burden differences between reproductive period of and post-reproductive of Père David’s deer. An asterisk (*) means significant differences.

In addition, the diversity of parasites was significantly higher during the reproductive period of Père David’s deer than during the post-reproductive period (Fig. 4, GLMM, *Z* = 2.456, *df* = 1152, *p* = 0.014). The statistical values and model effect results are shown in Table 5. There were no significant seasonal differences in the prevalence (GLMM, *F* = 1.801, df1=1, df2=1152, *p* = 0.180, strongyles; *F* = 1.834, df1=1, *df* = 1152, *p* = 0.176, *Eimeria* spp.; *F* = 1.758, df1=1, df2=1152, *p* = 0.185, *Moniezia* spp.; *F* = 1.331, df1=1, *df* = 1152, *p* = 0.249, *Trichuris* spp.). There were no significant seasonal differences in the burden of *Eimeria* spp. (LMM, *F* = 2.469, df1=1, df2=1152, *p* = 0.116). The burden of strongyles, *Moniezia* spp. and *Trichuris* spp. were significant higher during the reproductive period than post-reproductive period (LMM, *Z* = 2.135, *df* = 1152, *p* = 0.033, strongyles; *Z* = 2.234, *df* = 1152, *p* = 0.026, *Moniezia* spp.; *Z* = 2.305, *df* = 1152, *p* = 0.021, *Trichuris* spp.). The aggregation of Strongyles was higher during the reproductive period than they were during the post-reproductive period (K-S, *Z* = 1.167, *n* = 1154, *p* = 0.011).

**Table 5:** The differences of parasite parameters between reproductive period and post-reproductive period.

Item	Rep	Post-Rep	GLMM/LMM								
Sample number	296	858	F	df (n)	df (d)	P	Random	Estimate	Residual	Z	p
Diversity	0.5912 ± 0.04585	0.4674 ± 0.02193	6.034	1	1152	0.014	1	0.036	0.096	0.372	0.710
							2	0.045	0.083	0.546	0.585
Strongyles burden	88.51 ± 9.75	48.37 ± 3.10	4.557	1	1152	0.033	1	0.018	0.007	0.250	0.802
							2	0.028	0.058	0.476	0.634
Moniezia spp. burden	36.49 ± 8.70	11.89 ± 1.96	4.992	1	1152	0.026	1	0.059	0.015	0.397	0.691
							2	0.011	0.047	0.233	0.816
Trichuris spp. burden	5.07 ± 1.45	2.10 ± 0.52	5.312	1	1152	0.021	1	0.045	0.038	0.119	0.905
							2	0.032	0.077	0.419	0.675

**Notes.**

Rep indicates reproductive period; Post-Rep indicates post-reproductive period.

## Discussion

In this study, summer demonstrated the highest parasite diversity. Higher parasite diversity yields more infections and higher mortality (Betts et al., 2018). If the parasite load and diversity increase simultaneously, the high diversity will have a negative impact on the host in most cases (Johnson & Hoverman, 2012). Summer also had the highest burden of parasites in this study, showing that the situation in summer is relatively poor.

In this study, the prevalence, parasite burden, and aggregation of strongyles, *Eimeria* spp., *Moniezia* spp., and *Trichuris* spp. showed similar trends, being highest in summer and lowest in winter, although only some of these differences were significant. There could be three reasons for this: high prevalence, parasite burden, and aggregation in summer. First, there are more sources of infection in summer; second, the chain of transmission in summer is smoother; and third, the status of the host in summer is more conducive to parasitic infection. These are outlined in the sections below.

### Parasite sources

In the habitat of the Chinese Père David’s deer population, the temperature gradually rises from spring to summer. Previous research has shown that, within an appropriate range, an increase in environmental temperature can increase the growth rates of pathogen populations (Woodhams et al., 2008). Temperature affects the development of larvae in faeces. These larvae are in the developmental stages, and higher temperatures cause the larvae to preferentially develop into adult stage (Viney & Lok, 2015). This means that higher temperatures will accelerate the parasite life history process. The infectivity of these parasite larvae increases with temperature, and higher temperatures yield higher cercarial production (Goedknegt et al., 2015). In this study, parasitic infection indicators showed a consistent trend with temperature, as more parasite eggs were detected in the faecal samples in summer than in the other seasons.

Summer is the season with the highest rainfall in China. In previous studies on springbok, a strong seasonal relationship was further related to monthly rainfall, with peaks in parasite prevalence and propagule shedding intensity occurring after peak rainfall (Turner, 2009). This suggests that a peak in parasitic infection can be expected shortly after the peak in summer rainfall. The results of this study seem to support this suggestion. There are also studies that suggest that warm, humid weather favours parasite egg persistence in faeces (Cossío-Bayúgar et al., 2015). Combining these two phenomena, there are more sources of parasites in summer.

### Parasite infection

The complete infection route includes the source, route of exposure, and host susceptibility. The second point is the route of exposure. The prerequisite for certain parasites to infect the host is to develop into a infective larvae or be ingested by an intermediate host. The results showed that the parasites successfully infected in summer are the highest, which means summer conditions are more suitable for parasites to complete their life cycle. Among the several parasite groups, except for *Moniezia* spp. (the life cycle includes mites as an intermediate host, and then the infected mites are ingested by ruminants (Diop et al., 2015)), the rest had a faecal–oral life cycle (Dubey & Jenkins, 2018; Gruner & Cabaret, 1985; Nejsum et al., 2012). Rainfall and temperature significantly affect the development, transmission, and infection rates of parasites (Altizer et al., 2006; Nielsen et al., 2007; Turner & Getz, 2010). In the wet season, the increased temperature and humidity may enhance *Eimeria* spp. transmission (Turner, 2009). Parasites with faecal–oral transmission routes that results in seasonal variation in the probability of host exposure to parasites (Gorsich, Ezenwa & Jolles, 2014). *Moniezia* spp. require relatively warm temperatures for approximately one to three months to develop into cysticercoids within their intermediate hosts (Narsapur & Prokopic, 1979). In other words, suitable conditions in summer are conducive to the spread of parasites. In addition, summer is the most prolific season for plants, and it is a good time for herbivores to supplement their nutrition. But the infective stage of strongyles will be on the grass and waiting for the host to intake (Gruner & Cabaret, 1985). The consumption of plants by herbivores is a common route of parasite transmission (Ramnath, 2009). Summing up, the chances of the host being exposed to the parasite will increase, which means the parasites have a relatively greater chance of contacting the host in summer.

### Host status

Summer is the main reproductive period of Père David’s deer (Li et al., 2001). The following four reasons may cause more parasitic burden during the breeding period of Père David’s deer. (1) Trade-offs between reproductive efforts and immunity; (2) Hormone-induced immunosuppression; (3) Parasitised mates increase infection risk for partners. (4) New-born susceptibility.

(1) Trade-offs between reproductive efforts and immunity

In springbok, an increase in stress related to reproduction was linked to a decrease in immunity (Turner et al., 2012) and a positive correlation between reproductive effort and parasitism has been found in several studies (Corlatti et al., 2012; De la Peña et al., 2020a; Pelletier et al., 2005). The reproductive activities of Père David’s deer include rutting, courtship, competing for mates, fighting, chasing stags, mounting other stags, sex hormone secretion, gestation, lactation, and nursing, among others (Cizauskas et al., 2015; Corlatti et al., 2012; Li et al., 2001). These breeding efforts consume resources and energy (Blaxter, 1989; De la Peña et al., 2020b; Lappan, 2009; Meylan et al., 2013) leading to trade-offs between reproduction and immunity. Immunity against parasites is of relatively low priority when compared with maintenance and reproduction (Carlton, Cooper & Demas, 2014; Coop & Kyriazakis, 1999). The result is that reproductive efforts can suppress immune functions, thereby increasing parasitic infection (Nordling et al., 1998).

(2) Hormone-induced immunosuppression

Père David’s deer secretion of sex hormones, such as testosterone and progesterone, mainly occurs in summer (Li et al., 2001), which is the main Reproductive period of Père David’s deer. Combining the results of previous studies and ours showed that the peak period of parasite infection and the reproduction period are consistent. Testosterone and progesterone can suppress immunity by regulating a series of physiological adjustments and cell activities (Hernández-Bello et al., 2010; Jones et al., 2000; Klein, 2004; Klein & Roberts, 2010; McKay & Cidlowski, 1999; Trigunaite, Dimo & Jørgensen, 2015). House wrens (*Troglodytes aedon*) eggs treated with testosterone had weakened anti-bacterial ability after hatching (Clairardin et al., 2011); progesterone stimulates the proliferation rate in some kinds of parasites (Morales-Montor et al., 2004), progesterone, contribute to either susceptibility or resistance to parasitic disease during pregnancy (Vargas-Villavicencio, De Leon-Nava & Morales-Montor, 2009).

This means that sex hormones, in addition to being a reproductive effort and requiring a constant host resource barometer, can directly act on immunity by themselves. Previous studies have shown that sex hormones have a certain inhibitory effect on immunity and a certain promotion effect on parasite infection (Klein, 2004; Vargas-Villavicencio, De Leon-Nava & Morales-Montor, 2009). Therefore, the immunosuppressive induction of sex hormones is also one of the reasons for high parasite infection in summer (reproductive period).

(3) Parasitised mates increase infection risk for partners

During reproduction, breeding is one of the common social activities of animals, mates share time and space; therefore, there is a significant risk of parasite transmission between the mates (Martinez-Padilla et al., 2012). Furthermore, Père David’s deer exhibit urine-sniffing behaviour (Li et al., 2001) during rut. As the soil and faeces sprayed with urine may contain eggs or larvae, this can greatly increase the chance of partners being exposed to parasites.

(4) New-born susceptibility

The gestation period of Père David’s deer is 270–280 days, and the calves are born from April to May. Around 60 days after birth, the calves start to eat a small amount of grass and they are weaned around 90 days (Li, Chen & Tang, 2008). There could be many parasite eggs and larvae on the grass, making it easy for the Père David’s deer calves to ingest them. New-born individuals are highly susceptible to infection owing to their undeveloped immunity, which leads to a sharp increase in the proportion of susceptible individuals in summer (Turner & Getz, 2010).

Based on these four reasons, the Père David’s deer are especially susceptible during the reproductive period (summer).

Dongting had the highest parasite diversity of the four regions. The prevalence, parasite burden, and aggregation of strongyles, *Eimeria* spp., *Moniezia* spp., and *Trichuris* spp. were generally highest in Dongting. The temperatures of the regions were similar, but the rainfall was different, which could indicate that the parasitic infection status was vulnerable to the level of rainfall (Altizer et al., 2006; Turner & Getz, 2010). The parasite burden of *Moniezia* spp. in Dongting was significantly higher than it was in the other three regions. The life cycle of *Moniezia* spp. needs an intermediate host, a mite (Elliott, 1986). Increased precipitation significantly increases the parasite burden of mites (Wu et al., 2014). Therefore, as Dongting receives more rainfall, it is easier for *Moniezia* spp. to complete their life cycle, leading to higher prevalence and parasite burden.

In addition, strongyles showed significant differences between seasons and regions, *Moniezia* spp. showed significant differences between regions, and the aggregation of strongyles differed between the reproductive period of Père David’s deer and post-reproductive period. Although parasite aggregation is important for stabilising host–parasite dynamics (Wilson et al., 2002), hosts with a high burden of parasites had more parasite transmission potential than those with a low parasite burden or no parasites (Sherrard-Smith et al., 2015). In addition, the Père David’s deer founder population only constituted 18 individuals and the population’s ability to survive setbacks is relatively poor. Therefore, we cannot ignore the spread of diseases, including parasites, in Père David’s deer. Low aggregation and high burden were considered together that had greater transmission potential and can cause population instability (Newey, Thirgood & Hudson, 2004; Sherrard-Smith et al., 2015), that is a random distribution and high parasite infection of parasites within the host population that can lead to instability (Newey, Thirgood & Hudson, 2004). Low parasite aggregation coupled with high parasite burden means that the host–parasite relationship might be unstable, and that some individuals have higher transmission potential than others. In other words, there is a high risk of parasite outbreaks. Strongyles and *Moniezia* spp. had low aggregation but high parasite burden in Dongting; thus, more management and monitoring might be required in Dongting.

## Conclusion

The reports on the gastrointestinal parasites of Père David’s deer were limited. The Père David’s deer population was established from only a small number of individuals. Thus, the transmissible (parasitic) diseases merit further attention due to the relatively low genetic diversity of Père David’s deer. This research provides basic information of Père David’s deer intestinal parasites. We revealed the main intestinal parasite groups; made a basic clarification of the diversity, prevalence, burden and aggregation; determined the spatial and temporal regularity of the parasites; discussed the causes of seasonal and regional patterns of parasitic infections. These works will benefit the management of the deer and provided important information for supporting the scientific management. The summer season and the reproductive period had great potential for parasite transmission, and there is a high risk of parasite outbreaks in the Dongting Lake area. More management and monitoring might be required in times and sites which at risk.

##  Supplemental Information

10.7717/peerj.11335/supp-1 File S1 Raw data The fecal egg counts, the number of infected samples and how many types of parasites are infected in each sample.

## Additional Information and Declarations

### Field Study Permissions

The following information was supplied relating to field study approvals (i.e., approving body and any reference numbers): The collection of fecal samples was approved by the Beijing Milu Ecological Research CenterHubei Shishou Milu National Nature ReserveThe East Dongting Lake National Nature ReserveJiangsu Dafeng Milu National Nature Reserve.

### Data Availability

The following information was supplied regarding data availability: The raw measurements are available in the Supplementary File.
